# Supplementary material for: Photoperiodic control of the Arabidopsis proteome reveals a translational coincidence mechanism
Source: Mol Syst Biol. 2018 Mar 1;14(3):e7962. doi: 10.15252/msb.20177962 (PMC5830654; doi:10.15252/msb.20177962)
Supplement: Supplementary file 1 — Appendix [file MSB-14-e7962-s001.pdf]

Appendix

Photoperiodic control of the Arabidopsis proteome reveals a translational coincidence mechanism

Daniel D Seaton, Alexander Graf, Katja Baerenfaller, Anna Flis, Mark Stitt, Andrew J. Millar, Wilhelm Gruissem

Table of Contents

Photosynthesis light reactions 1
Isoprenoid and chlorophyll biosynthesis 1
Carbohydrate metabolism 2
Fatty acid metabolism 4
Sulfate assimilation and glucosinolate metabolism 4
References 5

In this Appendix changes in protein abundance across photoperiods are outlined for a selection of metabolic pathways. This provides additional information on the highly concerted changes in these pathways and behavior of specific proteins which were omitted from the main text due to space limitations, but which are considered of interest to the plant science community by the authors.

Photosynthesis light reactions

Concerted changes in protein abundance were observed across the electron transport chain as well as in the light harvesting and chlorophyll binding complexes (LHCII) surrounding PSII. Five out of nine detected PSI proteins increase in abundance in longer photoperiods. In the light harvesting complexes, the highly abundant LHCB1.3 and LHB1B1 proteins belonging to LHCII subunit B1, and all three proteins of the LHCII subunit 2 (LHCB2.1, LHCB2.2, LHCB2.3) were down-regulated in longer photoperiods while LCHB4.1 and LCHB4.3 were up-regulated (Fig 3C). The later are isoforms of the monomeric antenna protein CP29. They form a complex with LHCII and contribute to adapt light capture to changing light conditions, though they have been most studied in high-light stress rather than altered photoperiod (Tikkanen and Aro, 2012). It is noteworthy that with a 3.3 fold up-regulation in longer photoperiods LHC4.1 ranks number 13 on the list of proteins with the highest FC in the dataset (Table EV3). The observed changes in the abundance of light harvesting proteins match reports on the transcript level for plants grown in different photoperiods (Baerenfaller et al., 2015; Flis et al., 2016). Implications arising from these results are outlined in the discussion section of the main text.

Isoprenoid and chlorophyll biosynthesis

Isoprenoids have essential roles in photosynthesis, respiration, membrane fluidity and regulation of growth and development in plants. Our study reveals that the abundance of enzymes involved in the synthesis of isoprenoids is differentially regulated across different parts of the pathway, including reduced investment in chlorophyll synthesis in long photoperiods concomitant with reduced LHC protein abundance.

The common precursor of all isoprenoids, isopentenyl diphosphate (IPP), is synthesized by the mevalonate (MVA) pathway in the cytosol and the 2-C-methyl-D-erythritol 4-phosphate (MEP) pathway in plastids. The enzyme 3-hydroxy-3-methylglutaryl-CoA reductase (HMGR) catalyses a key regulatory step in the MVA pathway. HMGR activity is modulated by a range of endogenous signals and external stimuli (Vranová et al., 2013). The abundance of HMGR1, which was shown to be the HMGR paralogue responsible for isoprenoid biosynthesis in leaves (Suzuki et al., 2009), was 2.6-fold higher in longer photoperiods indicating an increased flux through the MVA pathway. The opposite trend can be observed for the MEP pathway. In longer photoperiods the abundance of 1-deoxy-D-xylulose 5-phosphate synthase (DXS) and 4-hydroxy-3-methylbut-2-enyldiphosphate reductase (HDR) was significantly reduced. DXS and HDR are the main enzymes controlling flux through the MEP pathway (Rodríguez-Concepción, 2006). The observed reduction in enzyme abundance could reflect a lower demand of IPP for downstream pathways leading to diverse isoprenoids including chlorophyll, carotenoids, abscisic acid and plastoquinone. Several enzymes involved in carotenoid biosynthesis increase in longer photoperiods (Appendix Fig S10). However, IPP is synthesized in the chloroplast mainly during the day when the required reducing equivalents are available and flux through the MEP pathway was shown to be light dependent in several plant species (MongéLard et al., 2011). Plants growing in long days therefore have more time to synthesise IPP in the chloroplast, which might allow a lower investment and lower flux rate through the MEP pathway.

Carbohydrate metabolism

Enzymes related to primary carbon metabolism were broadly up-regulated in longer photoperiods as reflected by enrichment of the KEGG pathway of carbon fixation (ath00710), the TCA cycle (ath00020) and starch and sucrose metabolism (ath00500) in proteins with higher abundance in longer photoperiods (Table EV6; Appendix Figs S3, S4, S5).

The observed abundance changes of enzymes in primary carbon metabolism are highly orchestrated. This was especially pronounced for the TCA and Calvin-Benson cycles (Table EV6). In the KEGG database 60 enzymes are annotated as components of the TCA cycle (ath00020) of which 47 (78.3%) were quantified in the present study and 14 increased in abundance in long photoperiods (30% of quantified proteins). From KEGG pathway of carbon fixation (ath00710), which includes the Calvin-Benson cycle, 57 out of 69 annotated proteins (82.6%) were quantified, with 21 increasing with photoperiod (37%). The overview map provided by KEGG for these pathways shows that the up-regulated enzymes cover almost all reactions of these metabolic cycles (Appendix Fig S4).

In the KEGG annotated pathway of starch and sucrose metabolism 21 of 66 quantified proteins were identified as up-regulated in longer photoperiods. Sucrose is the major transport form of photosynthetically assimilated carbohydrates in plants to supply non-photosynthetic organs with carbon skeletons. Components of the whole sucrose metabolism including sucrose synthesis, transport and degradation were up-regulated in longer photoperiods (Appendix Fig S5 and Fig 4A). These enzymes include two sucrose phosphate synthase isoforms, sucrose phosphate synthase 1F (SPS1F) and 4F (SPS4F). SPSs catalyse the synthesis of sucrose phosphate from UDP-glucose and fructose-6-phosphate. SPS1F and SPS4F are thought to be the major SPSs in Arabidopsis. Loss of SPS1F or SPS4F function reduces maximal SPS activity by 80% and 13%, respectively (Sun et al., 2011). While SPS synthesize sucrose both during the day and the night, silencing of a SPS4F homolog

in tobacco inhibited starch degradation (Chen et al., 2005) indicating a specific role of this enzyme in sucrose synthesis at night. Sucrose is the main transport sugar exported from fully autotrophic source leaves to sink organs like young leaves, roots and flowers. The protein with the second highest (13.3-fold) increase in abundance in longer photoperiods is the sucrose transporter SWEET12 (Fig 4A). SWEET12, together with its isoform SWEET11, is most likely localized in the phloem parenchyma cells and release sucrose into the cell wall before sucrose is actively loaded to the phloem (Chen et al., 2012). We also observed up regulation of Hexokinase 1 (HXK1) in longer photoperiods. HXK1 catalyses the phosphorylation of glucose and functions as a glucose-sensor with central regulatory function in primary carbon metabolism (Smeekens, 1998) and a proposed role in the transcriptional regulation of photosynthesis, growth and senescence (Moore, 2003).

Further effects of photoperiod length on the starch synthesis pathway included the up-regulation of the plastid phosphoglucomutase (PGM1) (Fig 4A). PGM1 is essential for starch synthesis and regulates the partitioning of carbon into starch (Fernie et al., 2001). The abundance of starch synthase 1 (SS1) and starch branching enzyme 2.2 (SBE2.2) was also increased in long photoperiods. Both enzymes are not essential for starch granules formation but are required for amylopectin synthesis (Delvallé et al., 2005; Wattebled, 2005). Loss of function of these enzymes results in changed amylopectin chain length and thereby affects starch structure.

In the pathway of starch degradation, up regulation was observed for several key enzymes including phosphoglucan water dikinase (PWD), Like Sex Four 1 (LSF1) and Like Sex Four 2 (LSF2) (Fig 4B). All three enzymes are plastid localised, bind to the surface of starch granules and are essential for normal starch degradation (Stitt and Zeeman, 2012). PWD and LSF2 are part of the phosphorylation/de-phosphorylation cycle of glucose residues on the surface of the starch granule, which may regulate the starch degradation pathway (Scialdone et al., 2013). PWD specifically phosphorylates the C3 position of glucose residues following C6 phosphorylation by glucan water dikinase (GWD) (Ritte et al., 2006). LSF2 is a phosphatase with specific activity towards C3 phosphorylated glucose residues and catalyses the reverse reaction to GWD3 (Santelia et al., 2011). No catalytic activity has been shown for LSF1. However, LSF1, like LSF2 and GWD3 function, is essential for normal starch degradation (Comparot-Moss et al., 2010). Hence, LSF1 might play a regulatory role in the pathway or act as a scaffold protein to mediate protein-protein interactions.

Following phosphorylation of glucose residues on the starch surface, degrading enzymes, mainly β-amylases (BAMs) and debranching enzymes (DBEs) can access the granule and release short chained sugars (Stitt and Zeeman, 2012). BAM3 (also called CT-BMY) and isoamylase 3 (ISA3) had higher abundances in longer photoperiods (Fig 4B). ISA3 together with the β-amylases BAM3 and BAM1 are the main enzymes responsible for starch degradation in the mesophyll cells of Arabidopsis leaves. BAM1 did not show significant changes in abundance. The observed higher abundance of BAM3 and proteins involved in the cycle of glucan phosphorylation and dephosphorylation support a higher maximal rate of starch breakdown, consistent with the faster starch degradation during the night in long photoperiods (Baerenfaller et al., 2015; Sulpice et al., 2014).

The main breakdown product of starch is maltose, which is exported from the chloroplast via the maltose transporter (MEX1) and further metabolised in the cytosol by disproportionating enzyme 2

(DPE2) and an alpha-glucan phosphorylase 2 (PHS2). DPE2 and PHS2 protein abundance were significantly up-regulated in longer photoperiods (Fig 4B).

Fatty acid metabolism

Fatty acids are the building blocks of lipids and hence bio-membranes. They also have essential roles as storage compounds and carbon source during seed development. During plant growth the formation of new bio-membranes creates a high demand for phospholipids and fatty acids, thereby creating an important sink for newly assimilated carbon (Bao et al., 2000). Fatty acid catabolism besides being essential for lipid housekeeping is tightly interlinked with amino acid metabolism and hormone signalling (Baker et al., 2006).

We find that plants adjust the protein abundance of enzymes related to fatty acid metabolism to different photoperiods. In the KEGG pathway of fatty acid degradation significant enrichment was observed and 15 out of 31 proteins annotated in this pathway (ath00071) were up-regulated in long photoperiods (Table EV6, Appendix Fig S6). This indicates that plants in longer photoperiods have a higher capacity for beta-oxidation of fatty acids. Arabidopsis plants turn over approximately 4% of their total fatty acids in one diel cycle (Bao et al., 2000). On the other hand several enzymes involved in fatty acid biosynthesis are down-regulated in long photoperiods, including the biotin carboxylase CAC2, 3-ketoacyl-acyl carrier protein synthases I and III (KASI, KASIII) and enoyl-ACP reductase (MOD1) (Table EV6, Appendix Fig 7). Our results indicate that in long photoperiods Arabidopsis plants synthesise fatty acids over a larger fraction of the diel cycle and can therefore decrease the abundance of enzymes in this pathway compared to plants growing in short photoperiods. However, quantification of lipid level and the activity of enzymes in the fatty acid biosynthesis and degradation pathways would be required to strengthen these hypotheses.

Sulfate assimilation and glucosinolate metabolism

Sulfur is an essential component of the amino acids cysteine and methionine as well as part of co-enzymes, prosthetic groups and many secondary metabolites. Sulfate uptake and assimilation is tightly regulated in response to light as well as by integration of signals from carbon and nitrogen metabolism (Huseby et al., 2013; Kopriva, 2002; Koprivova et al., 2000). We find a reprogramming of sulfate assimilation in longer photoperiods. The first step in sulfate assimilation is the reduction of sulfate to adenosine-5-phosphosulfate (APS) catalysed by ATP sulfurylase. All four isoforms of the ATP sulfurylase (APS1 to APS4) had increased abundances in longer photoperiods (Fig 4C). APS is a substrate for primary and secondary sulfate assimilation. In the primary pathway APS is reduced by APS reductase and sulfite reductase to sulfide, which can be incorporated into the amino acid skeleton of O-acetylserine to form cysteine. Interestingly, several enzymes of this pathway including APS reductase 2 and 3 (APR2,APR3) as well as four cysteine synthases (ACS1, DES1, CS26 and CYSD2) are down-regulated in longer photoperiods (Fig 4C; Appendix Fig S8). In secondary sulfate assimilation APS is phosphorylated by APS kinase to 3-phosphoadenosine 5-phosphosulfate (PAPS). PAPS is the active sulfur donor for the synthesis of a variety of secondary metabolites. The abundance of APS kinase 1 (APK) increased in longer photoperiods.

The changes in abundance of sulfate assimilating enzymes indicate a shift from the synthesis of primary towards secondary sulfur-containing metabolites in longer photoperiods. This observation is

further supported by a concerted increase in abundance of enzymes involved in glucosinolate biosynthesis. Glucosinolates are sulfur-containing secondary metabolites that function in plant defence against pathogens. Out of 19 proteins annotated for the glucosinolate biosynthesis pathway in KEGG 12 were quantified in this study and 10 showed a significant increase in protein abundance in long photoperiods (Fig 4C, Table EV6; Appendix Fig S9). Accordingly, both the GO category and the KEGG pathway of glucosinolate biosynthesis (GO:0019761 and ath00966, respectively) were strongly enriched in the up-regulated proteins (Table EV2; Table EV6).

References

Baerenfaller, K., Massonnet, C., Hennig, L., Russenberger, D., Sulpice, R., Walsh, S., Stitt, M., Granier, C., Gruissem, W., 2015. A long photoperiod relaxes energy management in Arabidopsis leaf six. Curr. Plant Biol. 2, 34–45. doi:10.1016/j.cpb.2015.07.001

Baker, A., Graham, I.A., Holdsworth, M., Smith, S.M., Theodoulou, F.L., 2006. Chewing the fat: β-oxidation in signalling and development. Trends Plant Sci. 11, 124–132. doi:10.1016/j.tplants.2006.01.005

Bao, X., Focke, M., Pollard, M., Ohlrogge, J., 2000. Understanding in vivo carbon precursor supply for fatty acid synthesis in leaf tissue. Plant J. Cell Mol. Biol. 22, 39–50.

Chen, L.-Q., Qu, X.-Q., Hou, B.-H., Sosso, D., Osorio, S., Fernie, A.R., Frommer, W.B., 2012. Sucrose Efflux Mediated by SWEET Proteins as a Key Step for Phloem Transport. Science 335, 207–211. doi:10.1126/science.1213351

Chen, S., Hajirezaei, M., Börnke, F., 2005. Differential Expression of Sucrose-Phosphate Synthase Isoenzymes in Tobacco Reflects Their Functional Specialization during Dark-Governed Starch Mobilization in Source Leaves. Plant Physiol. 139, 1163–1174. doi:10.1104/pp.105.069468

Comparot-Moss, S., Kötting, O., Stettler, M., Edner, C., Graf, A., Weise, S.E., Streb, S., Lue, W.-L., MacLean, D., Mahlow, S., Ritte, G., Steup, M., Chen, J., Zeeman, S.C., Smith, A.M., 2010. A putative phosphatase, LSF1, is required for normal starch turnover in Arabidopsis leaves. Plant Physiol. 152, 685–697. doi:10.1104/pp.109.148981

Delvallé, D., Dumez, S., Wattebled, F., Roldán, I., Planchot, V., Berbezy, P., Colonna, P., Vyas, D., Chatterjee, M., Ball, S., Mérida, Á., D'Hulst, C., 2005. Soluble starch synthase I: a major determinant for the synthesis of amylopectin in Arabidopsis thaliana leaves: SSI mutant of Arabidopsis. Plant J. 43, 398–412. doi:10.1111/j.1365-313X.2005.02462.x

Fernie, A.R., Roessner, U., Trethewey, R.N., Willmitzer, L., 2001. The contribution of plastidial phosphoglucomutase to the control of starch synthesis within the potato tuber. Planta 213, 418–426. doi:10.1007/s004250100521

Flis, A., Sulpice, R., Seaton, D.D., Ivakov, A.A., Liput, M., Abel, C., Millar, A.J., Stitt, M., 2016. Photoperiod-dependent changes in the phase of core clock transcripts and global transcriptional outputs at dawn and dusk in Arabidopsis. Plant Cell Environ. 39, 1955–1981. doi:10.1111/pce.12754

Huseby, S., Koprivova, A., Lee, B.-R., Saha, S., Mithen, R., Wold, A.-B., Bengtsson, G.B., Kopriva, S., 2013. Diurnal and light regulation of sulphur assimilation and glucosinolate biosynthesis in Arabidopsis. J. Exp. Bot. 64, 1039–1048. doi:10.1093/jxb/ers378

Kopriva, S., 2002. Interaction of Sulfate Assimilation with Carbon and Nitrogen Metabolism in Lemna minor. PLANT Physiol. 130, 1406–1413. doi:10.1104/pp.007773

Koprivova, A., Suter, M., denCamp, R.O., Brunold, C., Kopriva, S., 2000. Regulation of Sulfate Assimilation by Nitrogen in Arabidopsis. Plant Physiol. 122, 737–746.

MongéLard, G., Seemann, M., Boisson, A.-M., Rohmer, M., Bligny, R., Rivasseau, C., 2011. Measurement of carbon flux through the MEP pathway for isoprenoid synthesis by 31P-NMR spectroscopy after specific inhibition of 2-C-methyl-d-erythritol 2,4-cyclodiphosphate

reductase. Effect of light and temperature: Carbon flux through the MEP pathway. Plant Cell Environ. 34, 1241–1247. doi:10.1111/j.1365-3040.2011.02322.x
Moore, B., 2003. Role of the Arabidopsis Glucose Sensor HXK1 in Nutrient, Light, and Hormonal Signaling. Science 300, 332–336. doi:10.1126/science.1080585
Ritte, G., Heydenreich, M., Mahlow, S., Haebel, S., Kötting, O., Steup, M., 2006. Phosphorylation of C6- and C3-positions of glucosyl residues in starch is catalysed by distinct dikinases. FEBS Lett. 580, 4872–4876. doi:10.1016/j.febslet.2006.07.085
Rodríguez-Concepción, M., 2006. Early Steps in Isoprenoid Biosynthesis: Multilevel Regulation of the Supply of Common Precursors in Plant Cells. Phytochem. Rev. 5, 1–15. doi:10.1007/s11101-005-3130-4
Santelia, D., Kötting, O., Seung, D., Schubert, M., Thalmann, M., Bischof, S., Meekins, D.A., Lutz, A., Patron, N., Gentry, M.S., Allain, F.H.-T., Zeeman, S.C., 2011. The Phosphoglucan Phosphatase Like Sex Four2 Dephosphorylates Starch at the C3-Position in *Arabidopsis*. Plant Cell 23, 4096–4111. doi:10.1105/tpc.111.092155
Scialdone, A., Mugford, S.T., Feike, D., Skeffington, A., Borrill, P., Graf, A., Smith, A.M., Howard, M., 2013. Arabidopsis plants perform arithmetic division to prevent starvation at night. eLife 2, e00669. doi:10.7554/eLife.00669
Smeekens, S., 1998. Sugar regulation of gene expression in plants. Curr. Opin. Plant Biol. 1, 230–234.
Stitt, M., Zeeman, S.C., 2012. Starch turnover: pathways, regulation and role in growth. Curr. Opin. Plant Biol. 15, 282–292. doi:10.1016/j.pbi.2012.03.016
Sulpice, R., Flis, A., Ivakov, A.A., Apelt, F., Krohn, N., Encke, B., Abel, C., Feil, R., Lunn, J.E., Stitt, M., 2014. Arabidopsis Coordinates the Diurnal Regulation of Carbon Allocation and Growth across a Wide Range of Photoperiods. Mol. Plant 7, 137–155. doi:10.1093/mp/sst127
Sun, J., Zhang, J., Larue, C.T., Huber, S.C., 2011. Decrease in leaf sucrose synthesis leads to increased leaf starch turnover and decreased RuBP regeneration-limited photosynthesis but not Rubisco-limited photosynthesis in Arabidopsis null mutants of SPSA1: Roles of SPS isoforms in photosynthesis. Plant Cell Environ. 34, 592–604. doi:10.1111/j.1365-3040.2010.02265.x
Suzuki, M., Nakagawa, S., Kamide, Y., Kobayashi, K., Ohyama, K., Hashinokuchi, H., Kiuchi, R., Saito, K., Muranaka, T., Nagata, N., 2009. Complete blockage of the mevalonate pathway results in male gametophyte lethality. J. Exp. Bot. 60, 2055–2064. doi:10.1093/jxb/erp073
Tikkanen, M., Aro, E.-M., 2012. Thylakoid protein phosphorylation in dynamic regulation of photosystem II in higher plants. Biochim. Biophys. Acta BBA - Bioenerg. 1817, 232–238. doi:10.1016/j.bbabio.2011.05.005
Vranová, E., Coman, D., Gruissem, W., 2013. Network Analysis of the MVA and MEP Pathways for Isoprenoid Synthesis. Annu. Rev. Plant Biol. 64, 665–700. doi:10.1146/annurev-arplant-050312-120116
Wattebled, F., 2005. Mutants of Arabidopsis Lacking a Chloroplastic Isoamylase Accumulate Phytoglycogen and an Abnormal Form of Amylopectin. PLANT Physiol. 138, 184–195. doi:10.1104/pp.105.059295

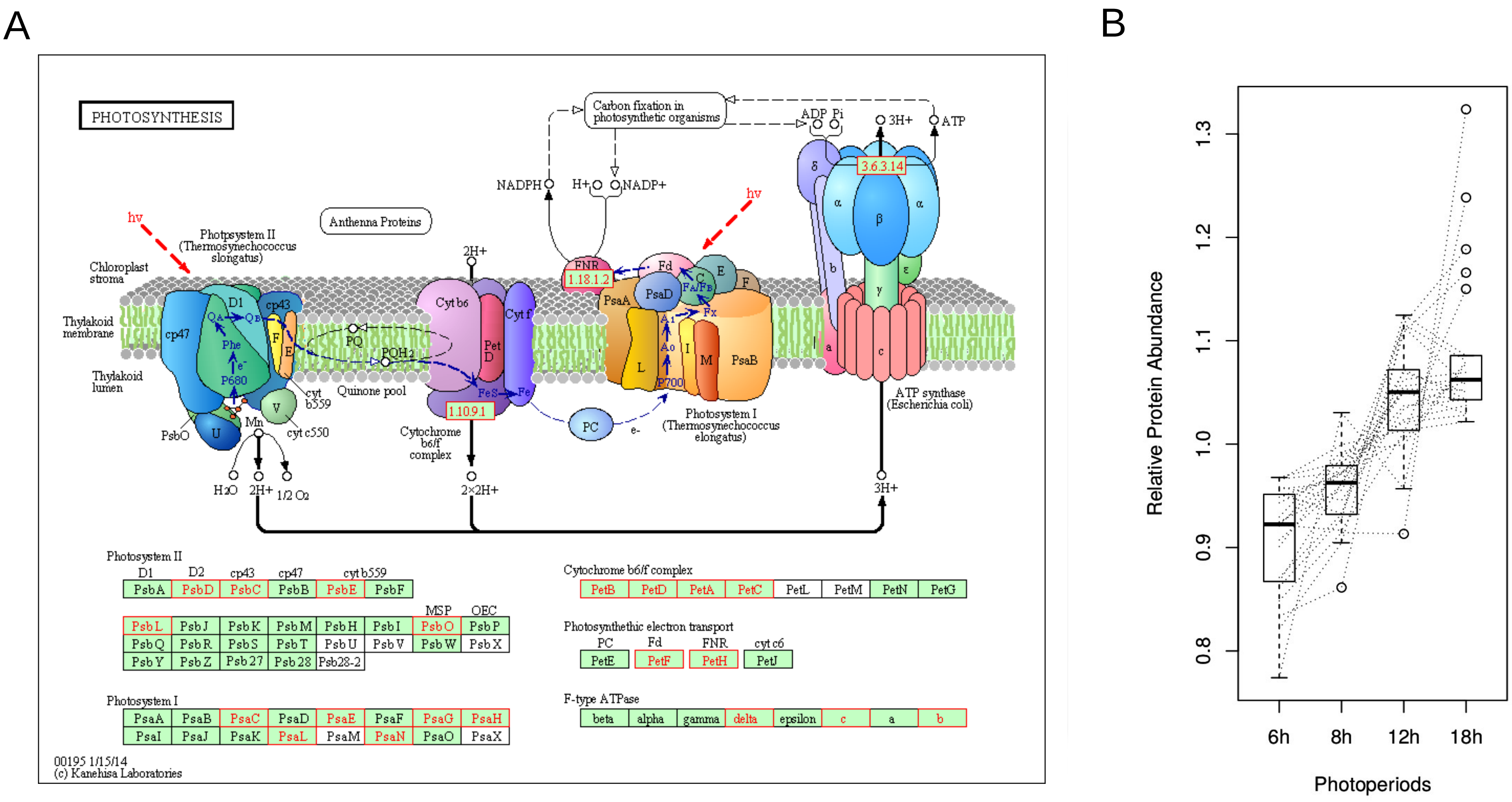

Appendix Figure S1. (A) KEGG photosynthesis pathway diagram. Quantified proteins are highlighted in green. Up-regulated proteins are further highlighted with red text. (B) Boxplot of protein abundance for the up-regulated proteins.

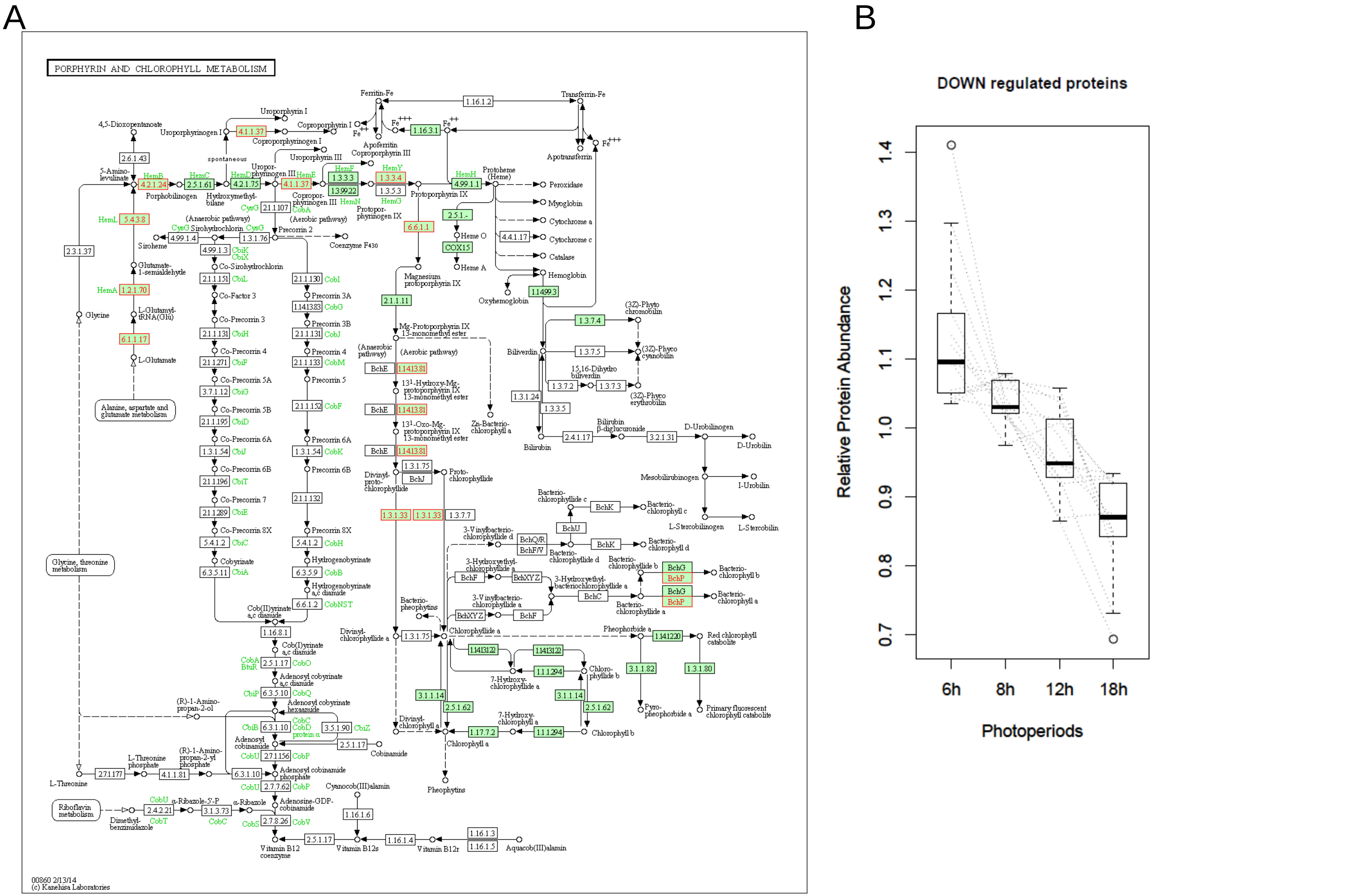

Appendix Figure S2. (A) KEGG porphyrin and chloropyll metabolism diagram. Quantified proteins are highlighted in green. Down-regulated proteins are further highlighted with red text. (B) Boxplot of protein abundance for the down-regulated proteins.

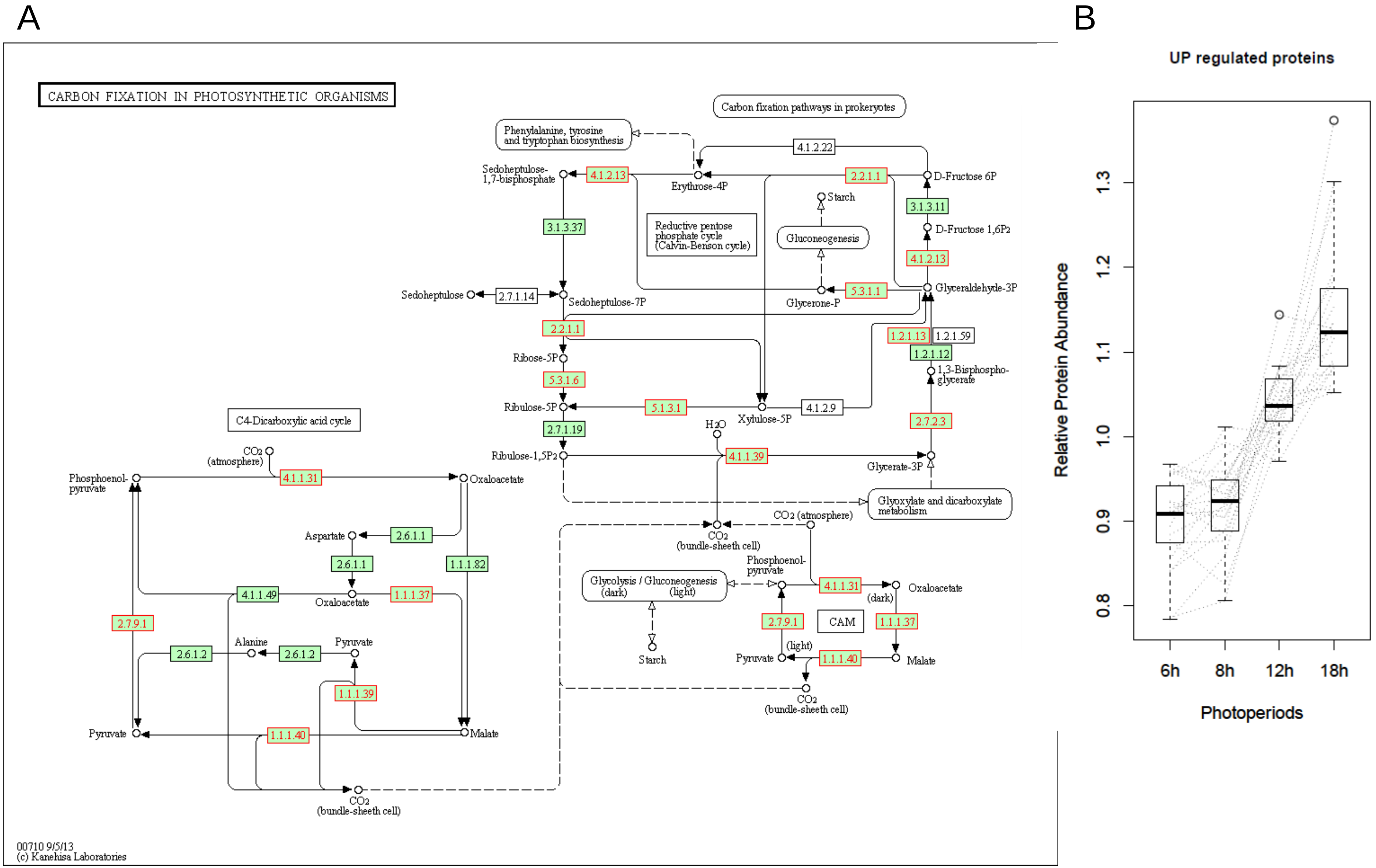

Appendix Figure S3. (A) KEGG carbon fixation diagram. Quantified proteins are highlighted in green. Up-regulated proteins are further highlighted with red text. (B) Boxplot of protein abundance for the up-regulated proteins.

A

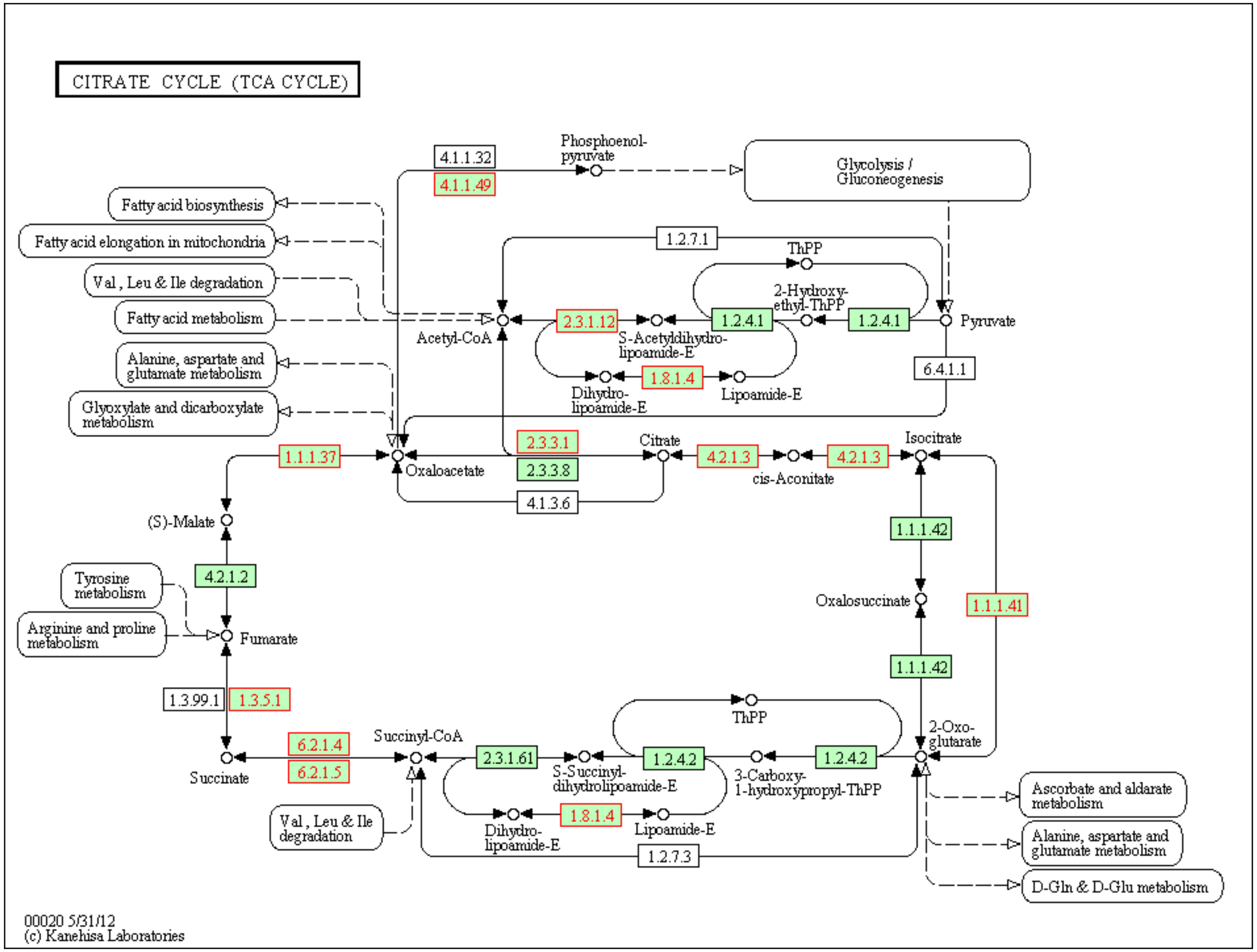

B

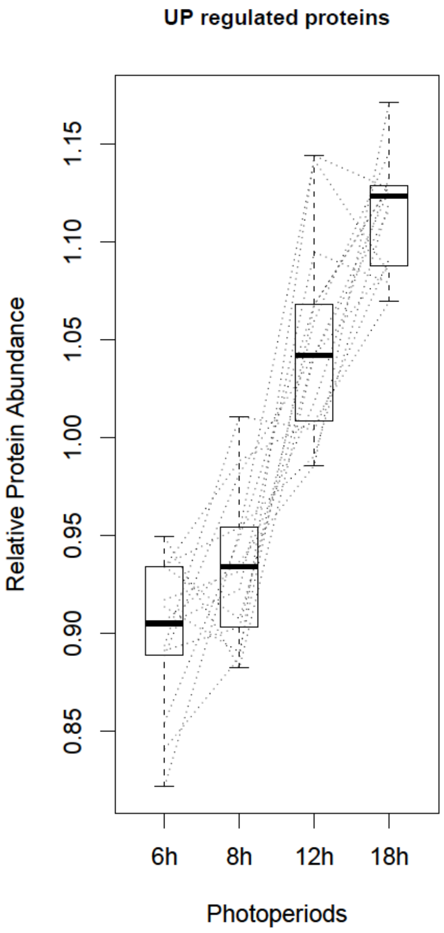

Appendix Figure S4. (A) KEGG TCA cycle diagram. Quantified proteins are highlighted in green. Up-regulated proteins are further highlighted with red text. (B) Boxplot of protein abundance for the up-regulated proteins.

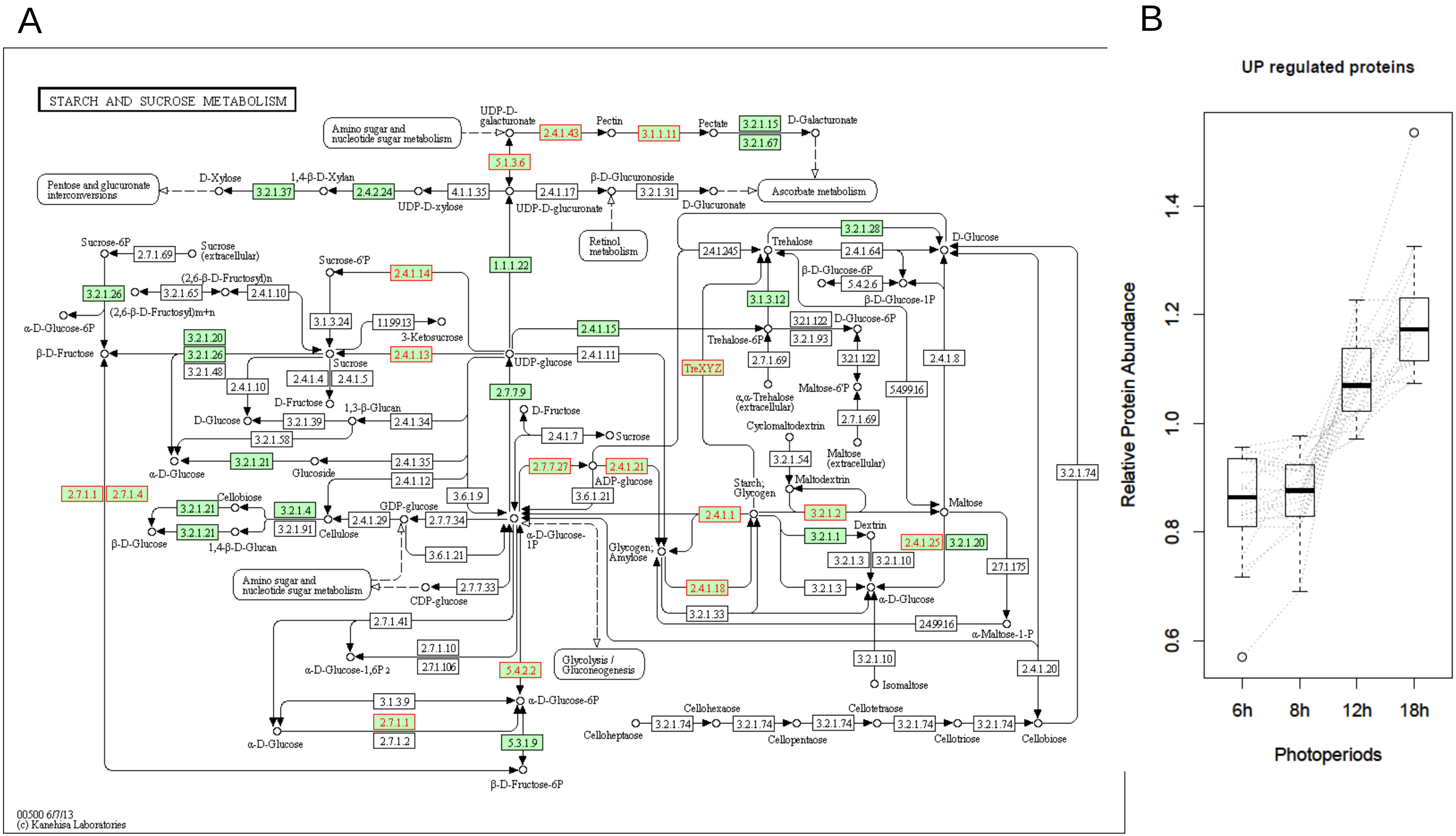

Appendix Figure S5. (A) KEGG starch and sucrose metabolism diagram. Quantified proteins are highlighted in green. Up-regulated proteins are further highlighted with red text. (B) Boxplot of protein abundance for the up-regulated proteins.

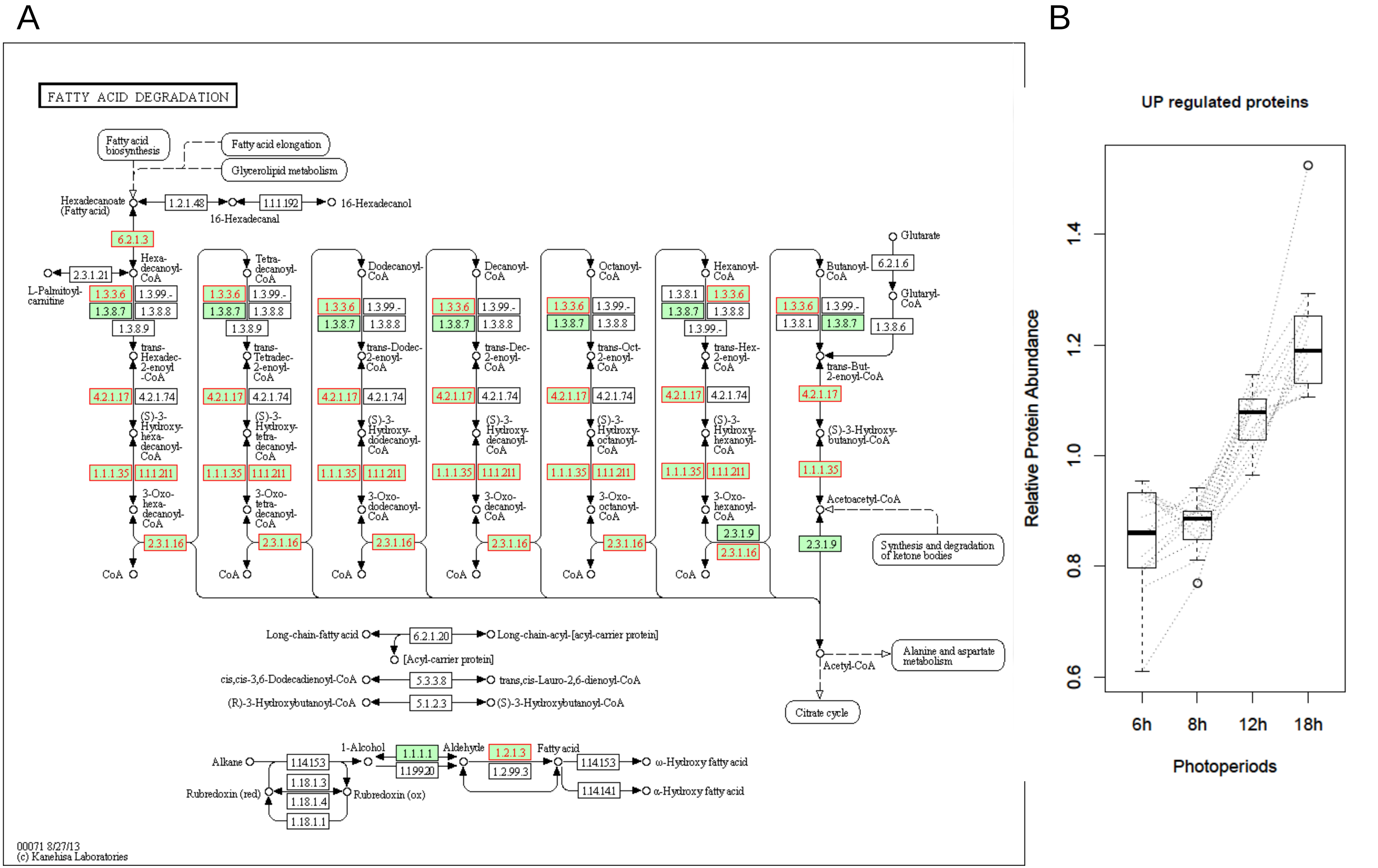

Appendix Figure S6. (A) KEGG fatty acid degradation diagram. Quantified proteins are highlighted in green. Up-regulated proteins are further highlighted with red text. (B) Boxplot of protein abundance for the up-regulated proteins.

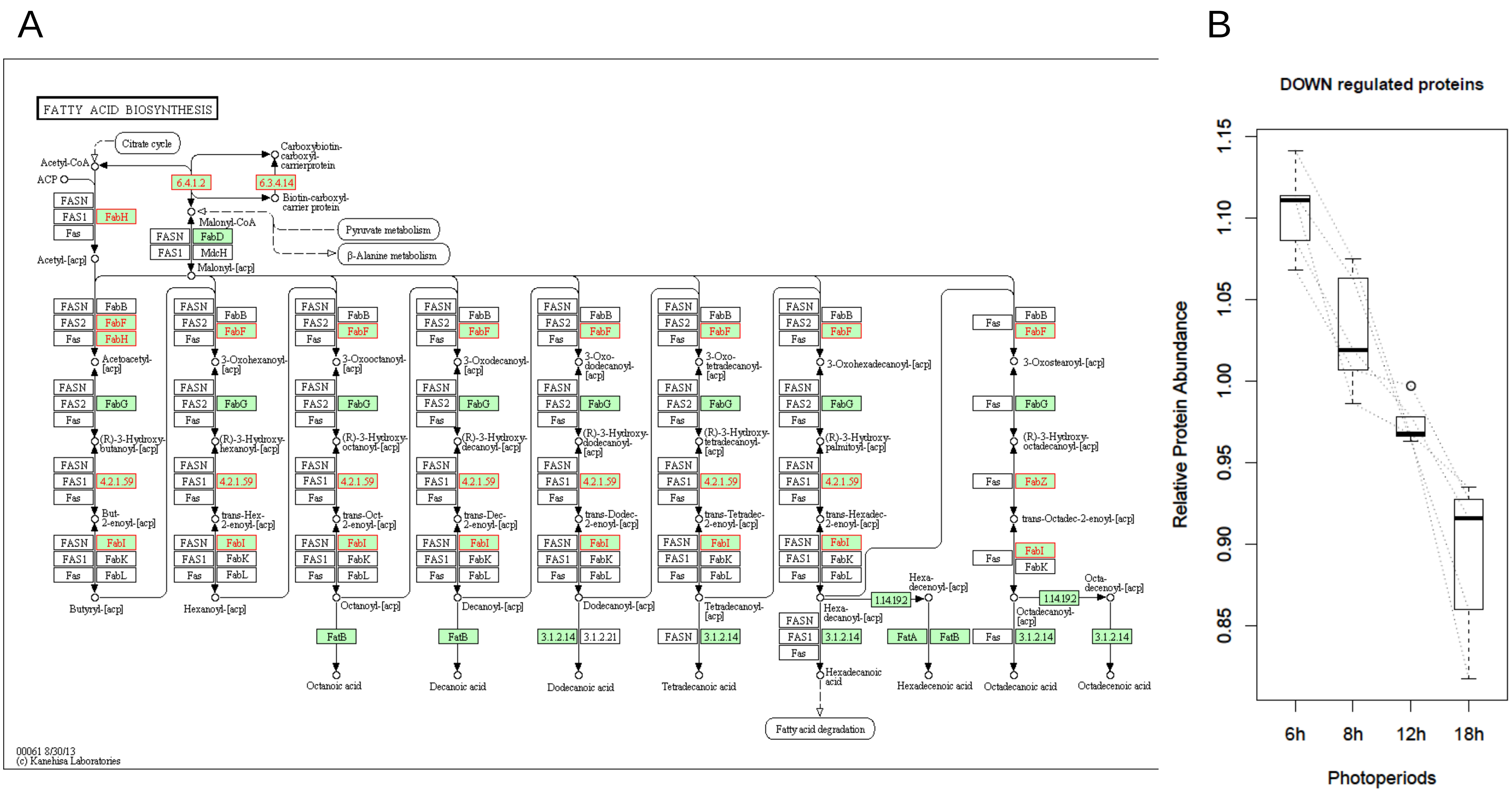

Appendix Figure S7. (A) KEGG fatty acid biosynthesis diagram. Quantified proteins are highlighted in green. Down-regulated proteins are further highlighted with red text. (B) Boxplot of protein abundance for the down-regulated proteins.

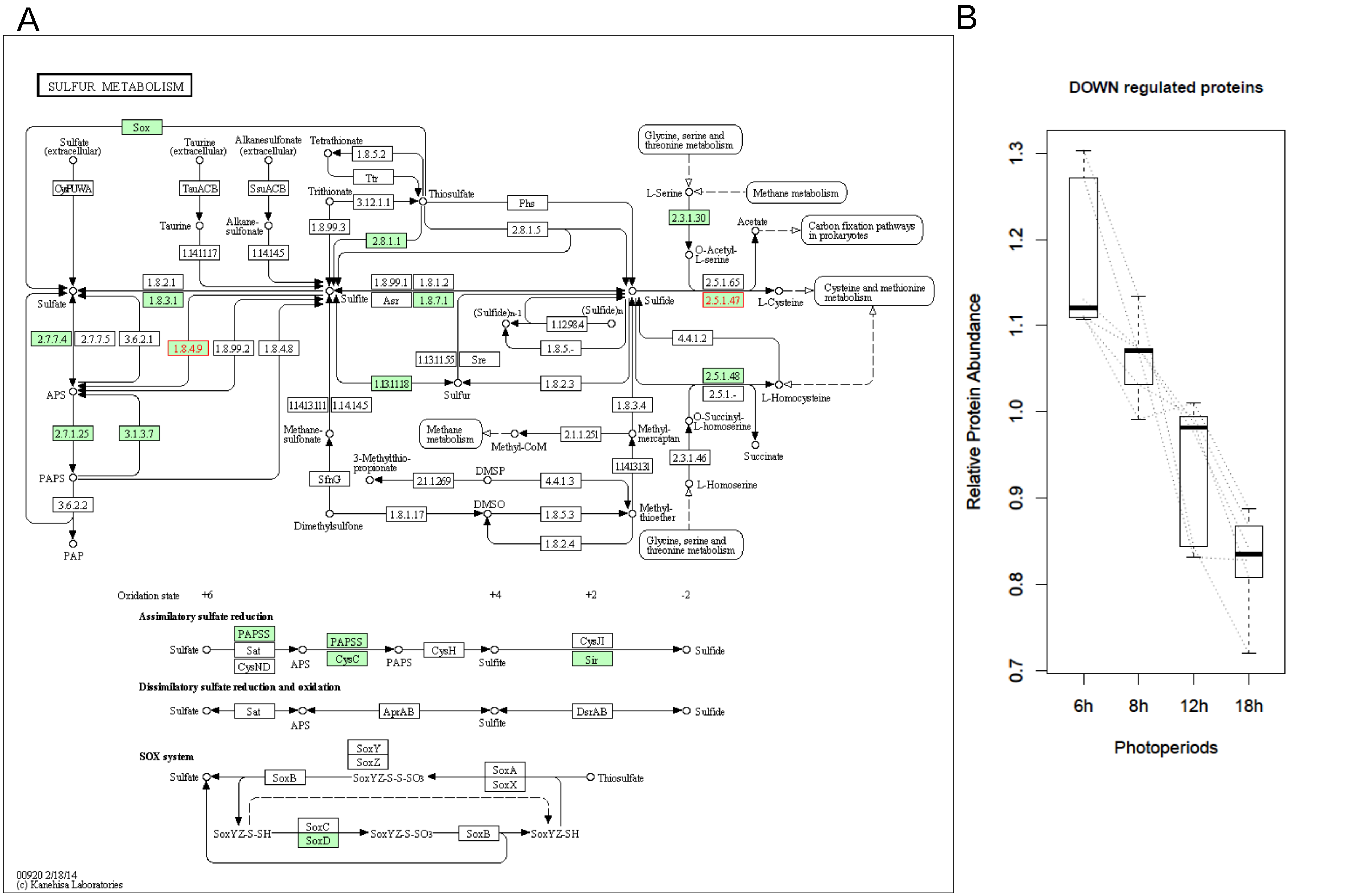

Appendix Figure S8. (A) KEGG sulfur metabolism diagram. Quantified proteins are highlighted in green. Down-regulated proteins are further highlighted with red text. (B) Boxplot of protein abundance for the down-regulated proteins.

A

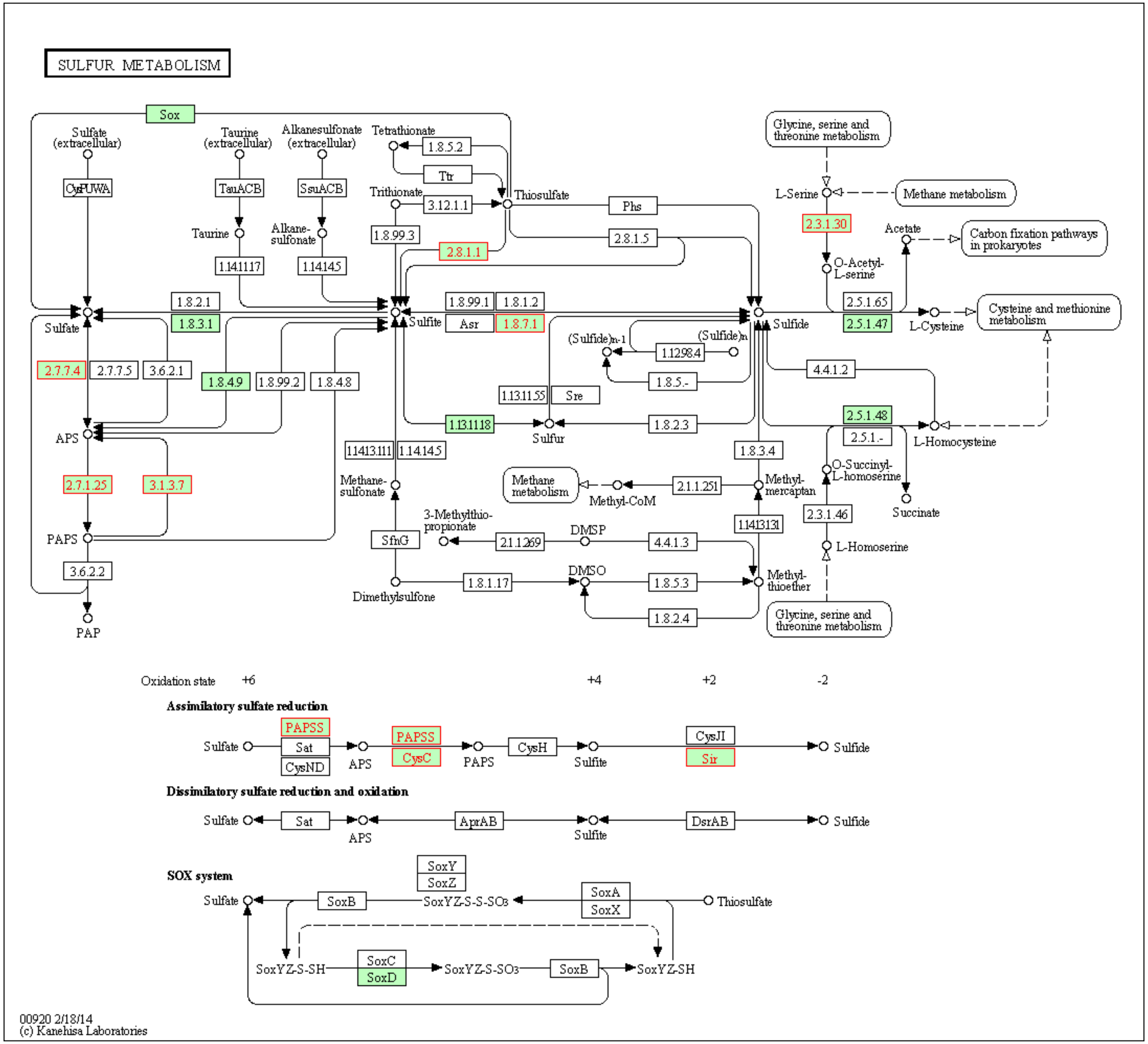

B

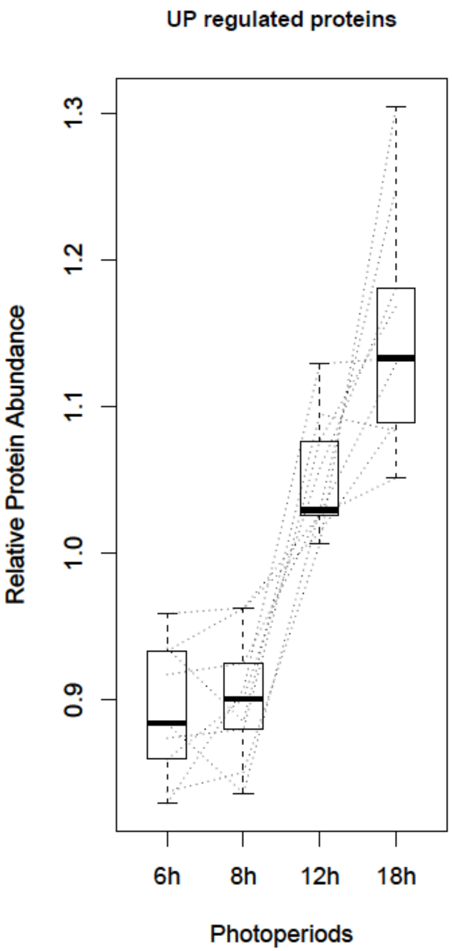

Appendix Figure S9. (A) KEGG sulfur metabolism diagram. Quantified proteins are highlighted in green. Up-regulated proteins are further highlighted with red text. (B) Boxplot of protein abundance for the up-regulated proteins.

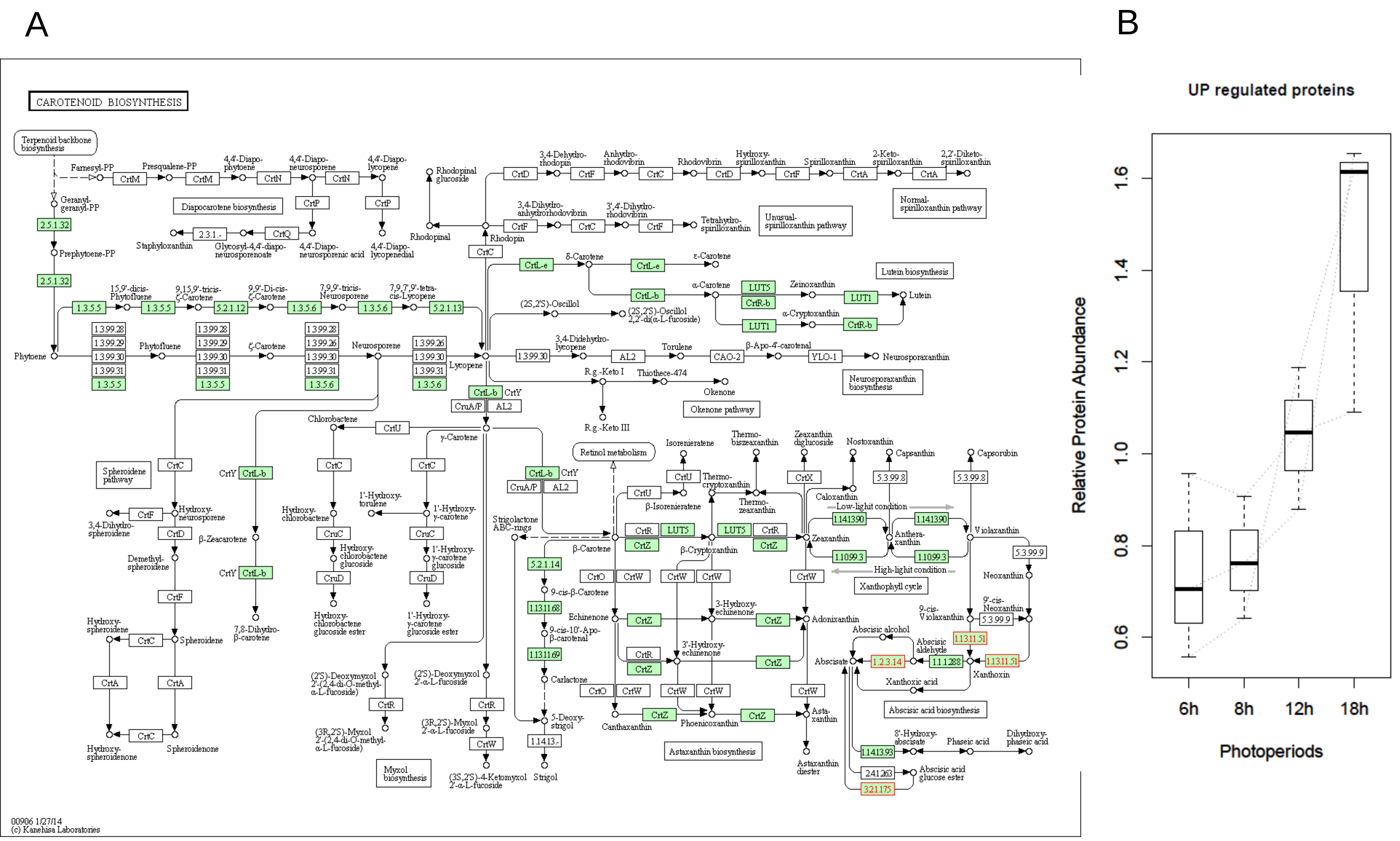

Appendix Figure S10. (A) KEGG carotenoid metabolism diagram. Quantified proteins are highlighted in green. Up-regulated proteins are further highlighted with red text. (B) Boxplot of protein abundance for the up-regulated proteins.
