# Supplementary material for: Photoperiodic control of the Arabidopsis proteome reveals a translational coincidence mechanism
Source: Mol Syst Biol. 2018 Mar 1;14(3):e7962. doi: 10.15252/msb.20177962 (PMC5830654; doi:10.15252/msb.20177962)
Supplement: Supplementary file 2 — Expanded View Figures PDF [file MSB-14-e7962-s002.pdf]

Expanded View Figures

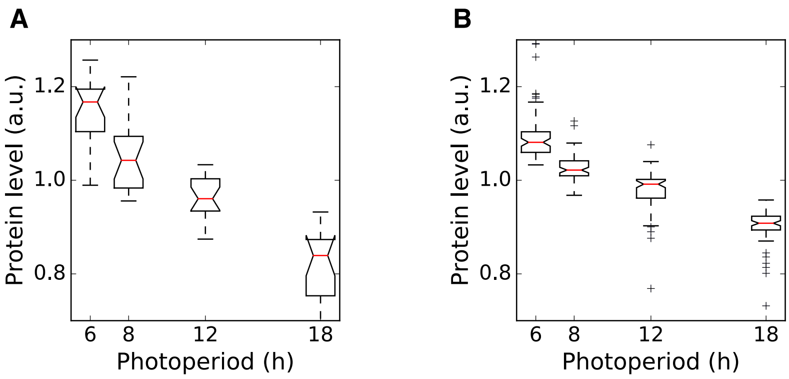

Figure EV1. Coordinated changes in ribosome biogenesis and ribosomal components.

A Changes in protein levels for 19 proteins in the KEGG pathway for ribosome biogenesis that decrease in abundance with increasing photoperiod length, with each protein normalised to its own mean level across all four photoperiods.

B As in (A), for 85 proteins in the KEGG pathway for ribosomes that decrease in abundance with increasing photoperiod length.

Data information: Boxes span the interquartile range. Whiskers span 1.5 times the interquartile range.

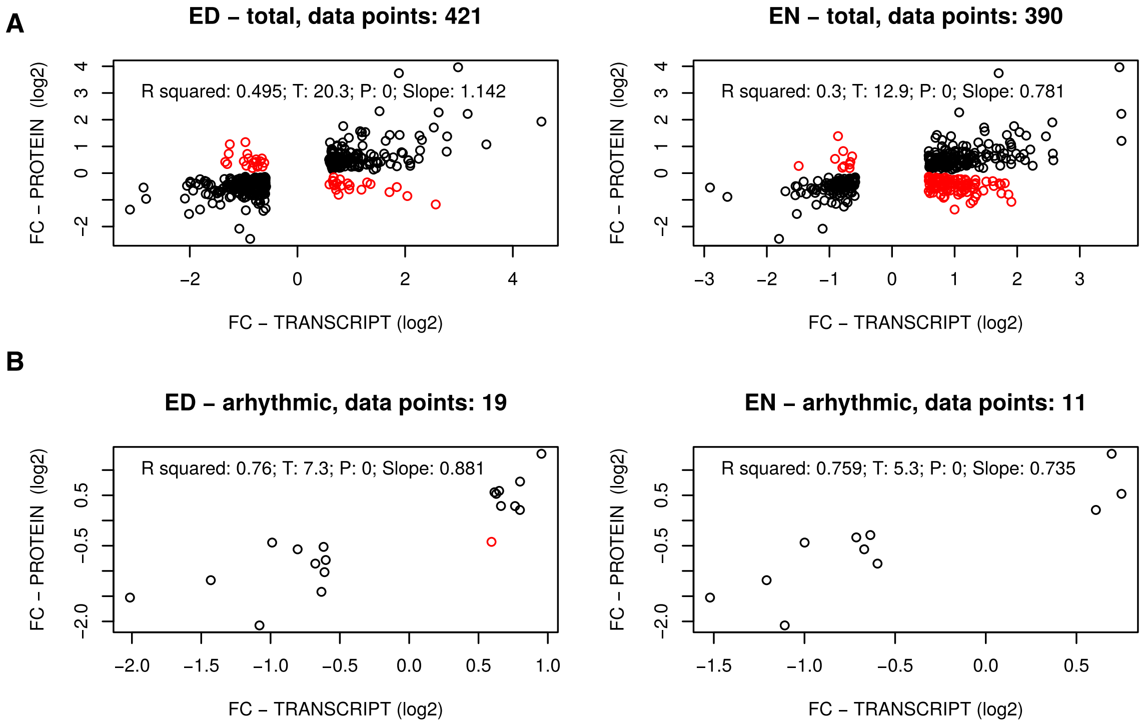

Figure EV2. Comparison of transcriptome and proteome photoperiod datasets.

A Correlations between the photoperiod proteome data and transcripts identified as exhibiting significant changes across photoperiods. Fold changes across photoperiods in transcripts and proteins are compared. Changing transcripts were identified at both ED and EN time-points (left- and right-hand panels, respectively), as described in Flis *et al* (2016). Fold changes are defined as the maximal fold change measured across photoperiods (Table EV3). Black dots indicate the direction of change was the same for both transcripts and proteins; red dots indicate the direction of change was different for transcripts and proteins. Transcript data are from samples taken from the same plants as were used for our proteomic analysis and were described in Flis *et al* (2016). The total number of transcript: protein pairs is given in the title of each graph.

B As in (A), for a subset of reliably arrhythmic transcripts (see Materials and Methods for the procedure used to identify arrhythmic transcripts).

© 2018 The Authors

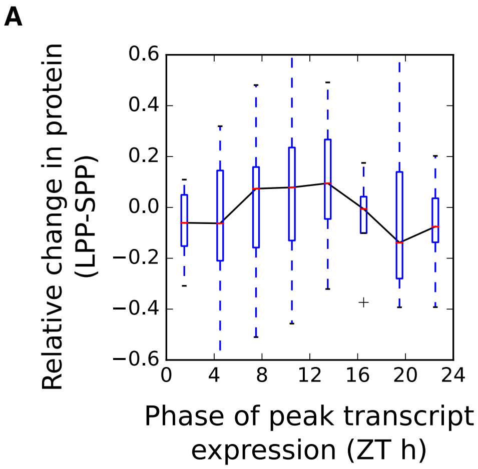

B

Leaf stage	1	2	3	4
ED	0.0007	0.033	0.024	0.026
EN	0.002	0.067	0.022	0.39

Figure EV3. Protein response to photoperiod in an independent dataset.

A Changes in protein levels between 8 h (SPP) and 16 h (LPP) photoperiods, as measured in Baerenfaller *et al* (2015), grouped according to the phase of peak transcript expression. Boxes span the interquartile range. Whiskers span 1.5 times the interquartile range.

B *P*-values of differences in protein levels for proteins across for leaf development stages (Baerenfaller *et al*, 2015) with evening-phased (ZT10 to ZT14, inclusive) and dawn-phased (ZT22 to ZT2, inclusive) transcripts (Bläsing *et al*, 2005), as calculated by Mann–Whitney *U*-test (note that in all cases the mean of the change from SPP to LPP was higher for the evening-phased group than the dawn-phased groups, as expected).

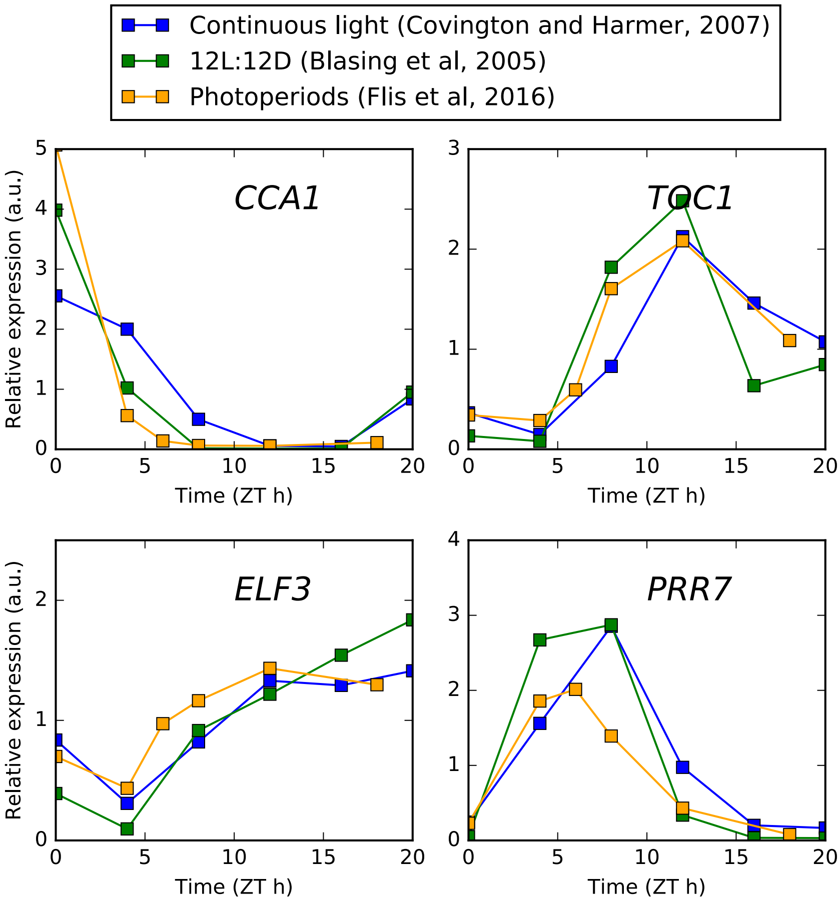

Figure EV4. Comparison of core clock transcript expression in different conditions.

Time series microarray data are plotted from experiments conducted in continuous light (Covington & Harmer, 2007) and 12L:12D light:dark cycles (Bläsing *et al*, 2005), along with data from combined EN and ED samples across 4-, 6-, 8-, 12- and 18-h photoperiods (Flis *et al*, 2016).

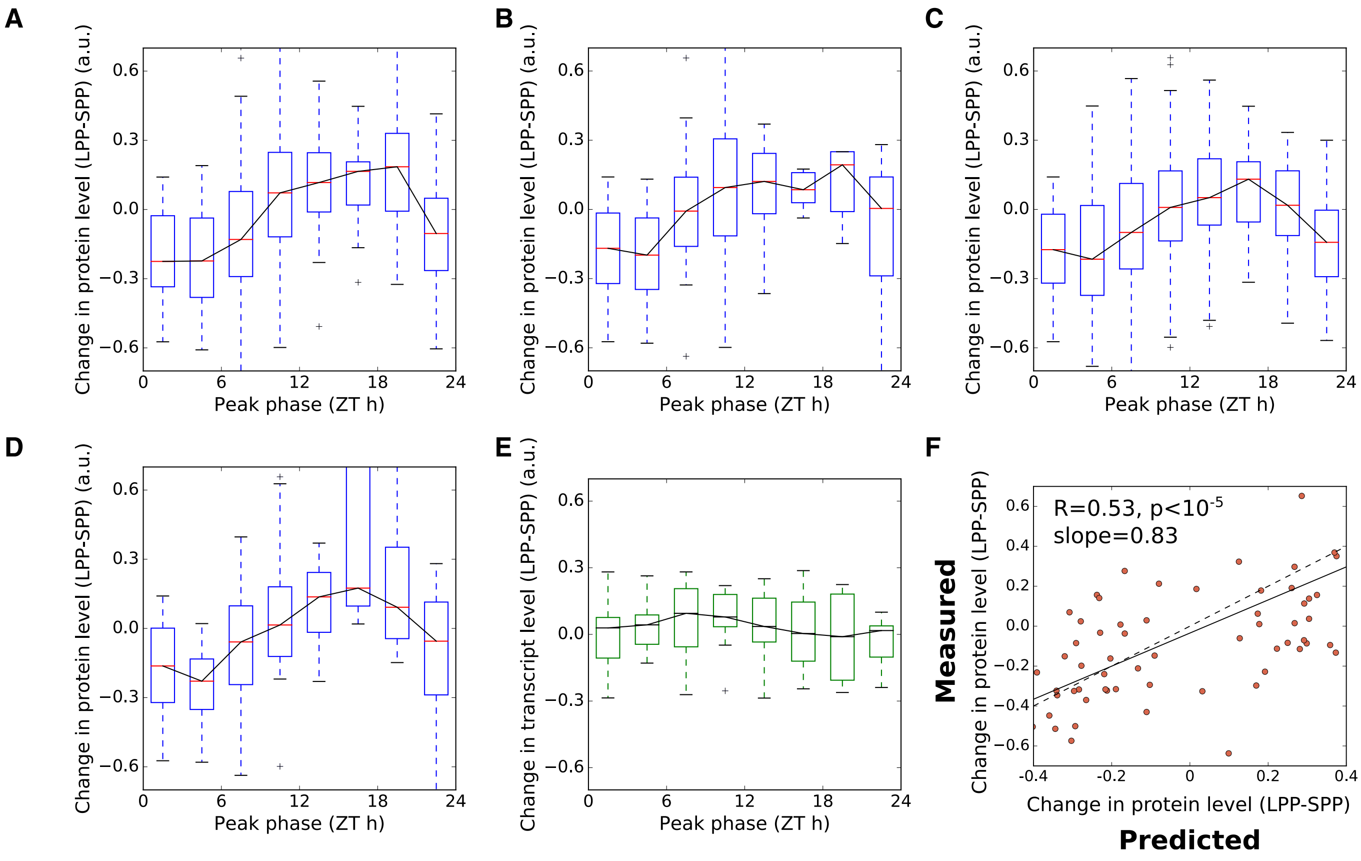

Figure EV5. Protein regulation with photoperiod after filtering for transcriptional regulation.

A Protein changes between 6-h (SPP) and 18-h (LPP) photoperiods, grouped by phase of peak expression, for the subset of 341 transcripts without changing levels across photoperiods, as judged by a comparison with the photoperiod microarray dataset of Flis *et al* (2016) (see text for details).

B As in (A), for the subset of 142 transcripts predominantly controlled by the circadian clock, as judged by a comparison with the circadian microarray dataset of Covington and Harmer (2007) (see text for details).

C As in (A), for the subset of 378 transcripts without changing levels between 8-h and 16-h photoperiods, as judged by a comparison with the microarray time series dataset of Michael *et al* (2008a,b) (see text for details).

D As in (A), for the 104 transcripts in the intersection of the subsets shown in (A–C).

E Changes in mean abundance of transcripts between 8-h (SPP) and 16-h (LPP) photoperiods in the microarray time series dataset of Michael *et al* (2008a,b), grouped by phase of peak expression, for the 104 transcripts in (D).

F Comparison of model to data, for the subset of transcripts that passed the filters in (A–C), for changes between 6-h and 18-h photoperiods (LPP-SPP), as in Fig 6E. Changes are plotted as differences between photoperiods, normalised to the mean. The dashed line indicates the case where model predictions match measured values. The solid line indicates the linear fit to the plotted data.

Data information: Boxes span the interquartile range. Whiskers span 1.5 times the interquartile range.

© 2018 The Authors

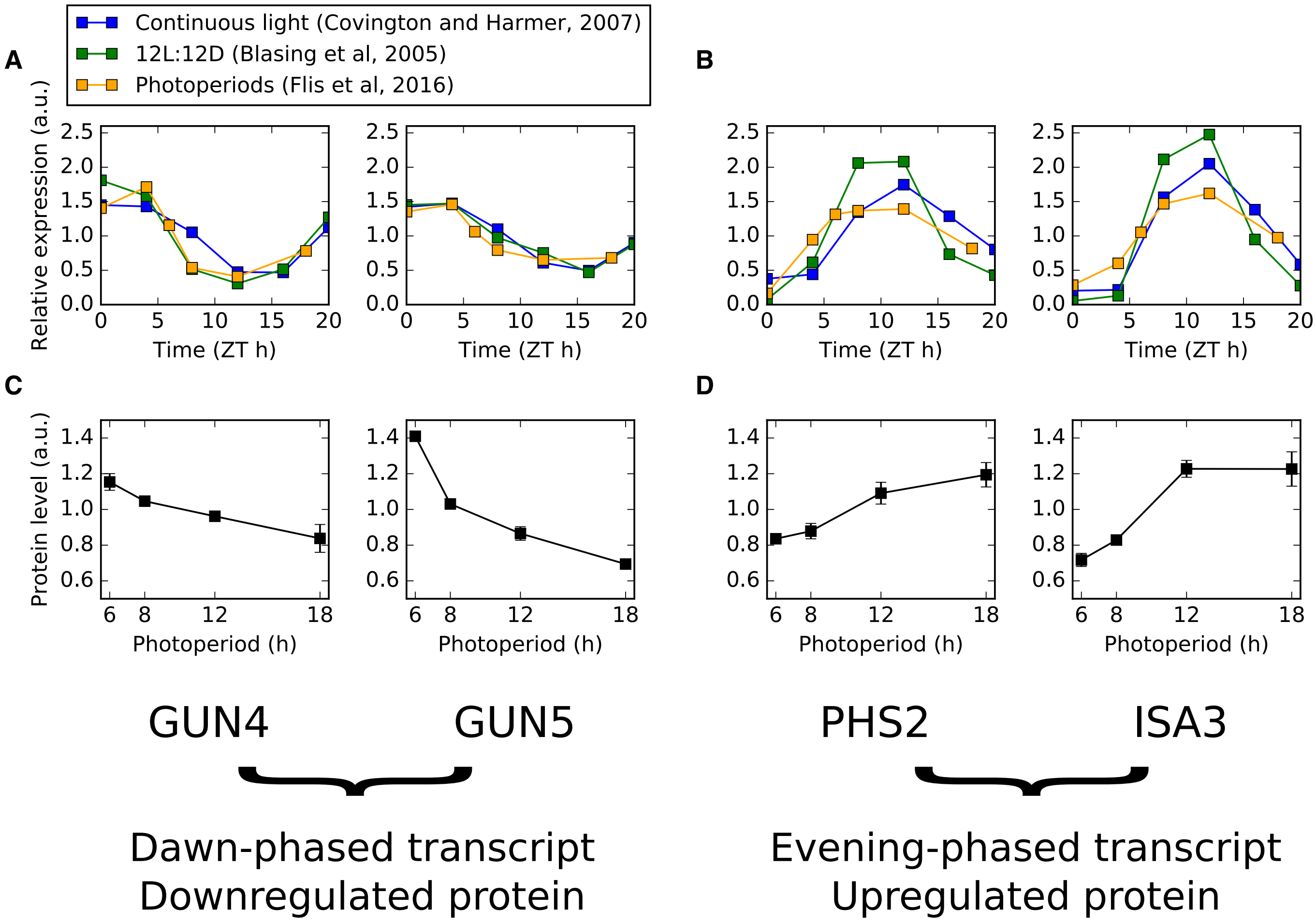

Figure EV6. Illustrative examples of photoperiod responses by translational coincidence.

A, B Gene expression for dawn-phased genes (GUN4, GUN5) are shown in (A) and evening-phased genes (PHS2, ISA3) are shown in (B) in multiple conditions, as measured by microarray. Time series data from experiments conducted in continuous light (Covington & Harmer, 2007) and 12L:12D light:dark cycles (Bläsing *et al*, 2005), along with pseudo-time series data from combined EN and ED samples across 4-, 6-, 8-, 12- and 18-h photoperiods (Flis *et al*, 2016). Data were mean-normalised.

C, D Protein abundance across photoperiods for transcripts quantified in (A, B), as quantified by mass spectrometry (this study). Error bars denote standard deviation (n = 3).

© 2018 The Authors

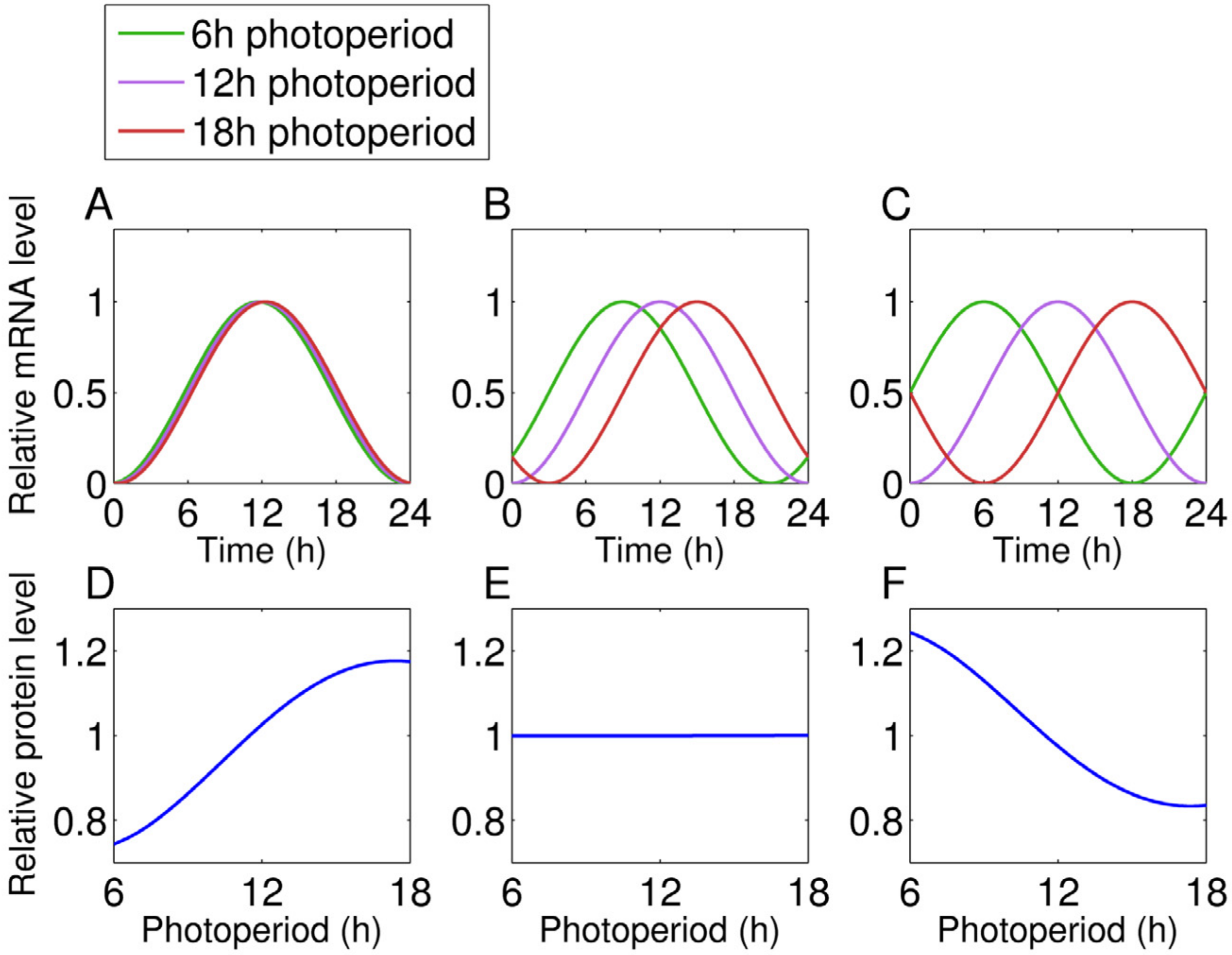

Figure EV7. Simulation of how clock responses affect the protein response to photoperiod.

A–C Clock-regulated transcript dynamics for a dawn-tracking (A), noon-tracking (B) and dusk-tracking (C) clock across three photoperiods. In each case, the transcript is expressed at ZT12 (i.e. dusk) in a 12-h photoperiod.

D–F Protein responses to photoperiod for the protein encoded by the transcript shown in A–C.

 © 2018 The Authors
